# Children’s digital privacy on fast-food and dine-in restaurant mobile applications

**DOI:** 10.1371/journal.pdig.0000723

**Published:** 2025-02-05

**Authors:** Christine Mulligan, Grace Gillis, Lauren Remedios, Christopher Parsons, Laura Vergeer, Monique Potvin Kent

**Affiliations:** 1 School of Epidemiology and Public Health, Faculty of Medicine, University of Ottawa, Ottawa, Ontario, Canada; 2 Munk School of Global Affairs & Public Policy, University of Toronto, Toronto, Ontario, Canada; Aga Khan University - Kenya, KENYA

## Abstract

Children are targeted by unhealthy food marketing on digital media, influencing their food preferences, intakes and non-communicable disease risk. Restaurant mobile applications are powerful platforms for collecting users’ data and are popular among children. This study aimed to provide insight into the privacy policies of top dine-in and fast-food mobile apps in Canada and data collected on child users. Privacy policies of the top 30 fast-food and dine-in restaurants in Canada were reviewed. A convenience sample of 11 English-speaking Canadian residents aged 9-12 years with fast-food apps on their mobile phones were recruited to use ≥1 fast-food restaurant mobile app(s). Children used the app(s) for 5-10 minutes and placed food orders. Parents submitted a Data Access Request (DAR) on their child’s behalf to the food company. Descriptive analysis and a flexible deductive approach to content analysis evaluated data collected through DARs. Overall, 26 privacy policies were analyzed. The intended age of app users was indicated by 12 (46%) food companies, 10 (39%) of which specified it as ≥13 years. No company had a compulsory age verification process. Twenty-four (92%) companies disclosed the data collected on app users: 23 (89%) did not distinguish between information pertaining to children or adults, and 21 (81%) described a protocol for action if they inadvertently collected data on children. Twenty-four DARs were sent to companies; 11 (45.8%) of which were fulfilled by companies, and 4 (16.7%) resulted in the receipt of children’s data. All responding food companies were found to collect sociodemographic information on child participants (e.g., name, email). Some collected other information, such as order details and available promotional offers. This study demonstrates current fast-food and dine-in restaurant privacy policies are insufficient and provides insight into data collected on children via fast-food apps. Policies must be strengthened to ensure children’s privacy and protection online.

## Author summary

Mobile apps owned by food companies can be a powerful means of collecting data on consumers, including children, and may be used to deliver personalized and targeted marketing to app users. In Canada, there are currently no policies that explicitly or intentionally aim to safeguard children’s data and privacy online or to protect children from targeted marketing of unhealthy foods. We analyzed the privacy policies and terms of service of the mobile apps of the top 26 fast-food and dine-in restaurants in Canada. We also examined what data these companies collect on children by recruiting 20 children aged 9–12 years to place food orders through companies’ fast-food restaurant mobile apps, and by asking parents to request information from the company about data collected on their child. Less than half of the companies provided this information, leaving it unclear what types of data restaurant companies are collecting on children or how the data is used. Most companies also did not specify the intended age of app users, nor did they have an age verification process. Our study highlights the need for federal regulations to protect children from unhealthy food marketing, including in food company mobile apps and other digital spaces.

## Introduction

The burden of overweight, obesity and non-communicable diseases (NCDs) remain a significant public health concern, including among children [1–6]. Diets high in ultra-processed foods and low in fruits and vegetables are a well-established risk factor for nutrition-related chronic diseases, such as obesity [7–9]. Marketing of unhealthy foods is an important contributor to poor diet quality in children [10–14]. Frequent exposure to marketing of foods high in sodium, sugars and/or saturated fat is associated with less healthy food preferences, purchase requests and intakes in children [10,12–16].

In Canada, children are exposed to a high volume of unhealthy food marketing instances across multiple platforms, including on digital media [17–20] through social media applications (e.g., TikTok, YouTube), company/brand websites or mobile applications, and mobile gaming apps, among others. [21]. In fact, Canadian children aged 7–16 years are exposed to an estimated 1560 to 9828 food ads per year on social media applications [20]. Companies are increasingly delivering digital marketing that is targeted to users, including children, based on their personal information and historical online behaviours [22]. This is concerning given that more than one-quarter of Canadian children in grades 4–11 report spending 1–2 hours per weekday and more than 3 hours per weekend day online [23]. Additionally, more than 70% of Canadian youth own a tablet or smartphone, and adolescents constitute the largest proportion of followers of frequently marketed food and beverage brands on social media [20,21,24]. In addition to social media, food company mobile applications also provide a means of collecting personal information and data about users’ food preferences and purchasing behaviours to deliver targeted marketing. These apps can be used to order food and beverages online and access exclusive deals and, while intended for a general audience, they are particularly popular among children. A recent survey of 1,341 Canadian children aged 9-12 years found that 65% of children reported having at least one restaurant app downloaded to their mobile device [25].

Besides bolstering marketing power and fostering user engagement, targeted marketing also raises concerns about children’s privacy. One study found that 67% of mobile apps used by preschool-aged children were frequently transmitting digital identifiers to third-party companies, thereby compromising children’s privacy [26]. Another study of more than 60,000 ‘kids’ apps’ found that most had potential privacy concerns, such as user-targeted marketing or linkages to users’ social media accounts [27]. Despite the rapidly expanding and evolving nature of mobile app development, there is a paucity of research concerning data handling and app commercial features that may compromise children’s digital privacy [28].

The WHO recommends that countries develop comprehensive policies to restrict unhealthy food marketing in all media and settings to which children are exposed, including digital media [16,29,30]. In the digital environment, children’s rights to privacy should be maintained without compromising their right to engage in online activity [22]. In Canada, there are currently no proposed or implemented food marketing policies that extend to the collection of personal information on digital media, including food company mobile apps. While Canadian regulations do require companies to obtain user consent for the collection of personal information (e.g., via a company privacy policy) [31], this information is often deceptive and confusing, particularly to children. In addition, Canadian law requires that companies provide users with access to their personal data and/or delete it upon request (or if requested by a parent/guardian on behalf of their child) [32].

Given the increasing amount of time children are spending online and the popularity of food company mobile apps with this age group, it is critical to understand how children’s digital privacy is being protected and what, if any, data are being collected about children. This study aims to provide insight into the privacy policies of the top fast-food and dine-in mobile apps in Canada, and determines the amounts and types of data collected on child users of some fast-food apps.

## Materials and methods

### Study design

To examine the privacy policies of the top fast-food and dine-in mobile apps in Canada, we conducted a policy scan and content analysis of fast-food and dine-in restaurant app privacy policies. Next, to determine the types of data that are collected by fast-food apps on children, we conducted a pilot observational prospective cohort study. The latter was approved by the University of Ottawa Research Ethics Board (approval number: H-01-23-8515).

### Fast-food and dine-in restaurants and their privacy policies

To understand what policies have been implemented by Canadian food and beverage companies to protect children’s digital data and privacy on their mobile apps, fast-food and dine-in restaurant privacy policies were reviewed and analyzed. Accordingly, the top 30 fast-food (i.e., where orders are placed at a counter) and dine-in restaurants (i.e., where orders and food service are provided tableside) by 2021 Canadian market share were identified [33] and those with company-owned mobile apps downloadable from the Canadian iOS App Store were selected for study (n = 27). Mobile apps were downloaded in November 2022 and searched for the presence of published privacy policies and/or terms of service agreements, which were available for 26 of the 27 companies (n = 21 fast-food restaurants, n=5 dine-in restaurants).

The privacy policies and terms of service agreements of the 26 sampled restaurants were downloaded and reviewed by a research assistant (GG). Data were extracted from these documents to respond to a series of questions adapted from previous research [34]. Questions covered topics such as information about the availability and features of the privacy policy, the data or personal information collected, the company’s approach to differentiating between child and adult users, the procedure to access personal data, and the security of the data (**S1 Table**). A flexible deductive content analysis approach was used to analyze the data, such that the data collection questions were used as the primary form but supplemented with additional relevant information as appropriate. A second research assistant (CM) reviewed the collected data to ensure completeness and accuracy. Data were summarized quantitatively by reporting the proportion of each response to all questions. Qualitative summaries of the privacy policy document content relevant to each question were also generated as needed.

### Pilot observational prospective cohort study

The apps of the top 5 foodservice chains were selected for this portion of the study, based on 2021 Canadian market share data [33]. All 5 chains were fast-food companies (e.g., as opposed to dine-in restaurants). A convenience sample of 11 English-speaking Canadian residents aged 9-12 years was recruited. Recruitment occurred through purposive sampling and word-of-mouth via posters sent out through the community networks of the research team and posted in community spaces (community Facebook groups, community centres). Given the exploratory nature of this study, the sample size was flexible as the primary concern was not the number of children, rather the number of Data Access Requests (DAR). This study aimed to analyze 3 or more DARs per mobile app included in the study (process outlined below), and 11 participants was sufficient to achieve this aim. To be eligible to participate, children were required to own a mobile device, and have at least one of the 5 selected fast-food company mobile apps downloaded to that device with an account already created in their name prior to subject recruitment. Informed consent was provided by the children’s parents/guardians, while the children provided informed assent.

**Fig 1:**
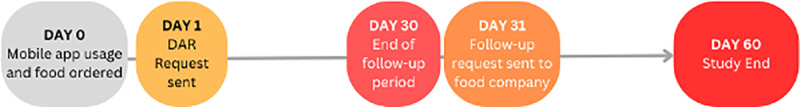
An overview of the study protocol and timeline for DARs.

Child participants were asked to use any of the 5 included fast-food mobile apps they had on their device for 5–10 minutes, including placing a mobile food order through the app. Children were able to use more than one fast-food company app as part of the study if the apps were already downloaded on their device prior to study recruitment. After a food order was placed, parent participants were instructed to submit a request for Data Access Request (DAR) to the company’s privacy office on behalf of their child, using an email template that was pre-drafted by the research team. Under the Personal Protection and Electronic Documents Act (PIPEDA), which governs how regulated private sector entities must handle Canadian residents’ personal information, individuals can access their data via DARs and companies are required to comply within 30 days (or provide reasons for refusing to provide the requested data) [31]. If parents did not receive a response from the company within 30 days of sending the DAR, they were asked to follow up with the company using another pre-drafted email template. If the DAR had been satisfied and the data was received, or if no data was received within 60 days of submitting the DAR (despite sending a follow-up email), data collection was considered complete. **Fig 1** provides an overview of the study protocol and timeline for DARs. Child participants received a $30 gift card of their choice as compensation, and were reimbursed for up to $20 of their food order placed through each fast-food app used as part of the study.

Descriptive analysis and a flexible deductive approach to content analysis were used to examine the participant data collected via DARs. Specifically, descriptive analyses examined the number and proportion of DARs that: were fulfilled; were fulfilled within 30 days; required additional following up; and resulted in receiving the children’s personal data. Reasons provided by companies for not fulfilling DARs were also examined. “Fulfilled” DARs included those where the food company replied to the request and either provided the data, deleted the data, or closed the child’s account. When no response was received from the company and there was no sharing of the data or deletion of the data or account, the DAR was considered unfulfilled. A series of questions adapted from previous research were used to guide the content analysis (**S1 Fig**) [34]. Using a flexible deductive approach, additional relevant information was used to supplement the data collection questions, as needed. The completeness and accuracy of data collection was verified by a second research assistant (CM). Data were summarized descriptively and presented overall and by food company, and company names were anonymized in the analysis.

## Results

### Fast-food and Dine-in restaurant privacy policies

**Table 1** summarizes the privacy policies and/or terms of service agreements for the mobile apps of Canada’s leading fast food and dine-in restaurants; a more detailed summary of the policies is provided in **S2 Table**. The policies of nearly half of the food companies (46.2%, n=12) specified the intended users of the mobile apps, most of which indicated the app was not meant for children under 13 years-old (38.5%, n = 10). Although some companies’ policies allowed users to voluntarily disclose their age (e.g., by entering their date of birth), other policies indicated that by setting up a profile on the app, the user confirmed they met the company’s minimum age requirement. No companies in this sample had a mandatory age verification procedure for app users.

**Table 1:** A summary of the mobile app privacy policies and/or terms of service agreements of the top 26 fast food & dine-in restaurants in Canada^1^.

Features of mobile app privacy policies and/or terms of service agreements		n (%)
Link to privacy policy on the app login, ‘make an account’ page, or homepage	Yes	26 (100)
	No	0 (0)
Indication of age of the intended app user	Yes	12 (46.2)
	No	14 (53.8)
Statement about procedure for handling data inadvertently collected on children	Yes	21 (80.8)
	No	5 (19.2)
Details of the specific kinds of personal information collected	Yes	24 (92.3)
	No	2 (7.7)
Distinction between information pertaining to children or adults	Yes	3 (11.5)
	No	23 (88.5)
Age verification process (e.g., entering date of birth)	Yes	0 (0)
	No	26 (100)
Requirement that certain information is provided, as a precursor to signing up for the service or acquiring products from the company	Yes	26 (100)
	No	0 (0)
Procedures for access and correction of information	Yes	22 (84.6)
	No	4 (15.4)

^1^Data collected *in* November 2022.

Most food companies (92.3%, n = 24) indicated the type of personal information collected by their mobile apps (e.g., name, email address, purchase history), but 88.5% (n = 23) did not distinguish between child and adult users. All the companies (100%, n = 26) specified that it was mandatory to provide personal information to create an account and use their mobile app services.

The privacy policies and/or terms of service agreements of 80.8% of food companies (n = 21) outlined a procedure for managing personal data invertedly collected on children under 13, including deleting the data without request (n = 11, 42.3%) or following a request from the child’s parent (n=9, 34.6%). Processes for accessing or correcting users’ personal data were specified by 57.7% (n=15) of food companies. No companies required payment to access participants’ data. Most companies (n = 25, 96.2%) provided contact information for privacy officers where inquiries regarding company privacy policies should be directed.

### Pilot observational prospective cohort study

**Fig 2:**
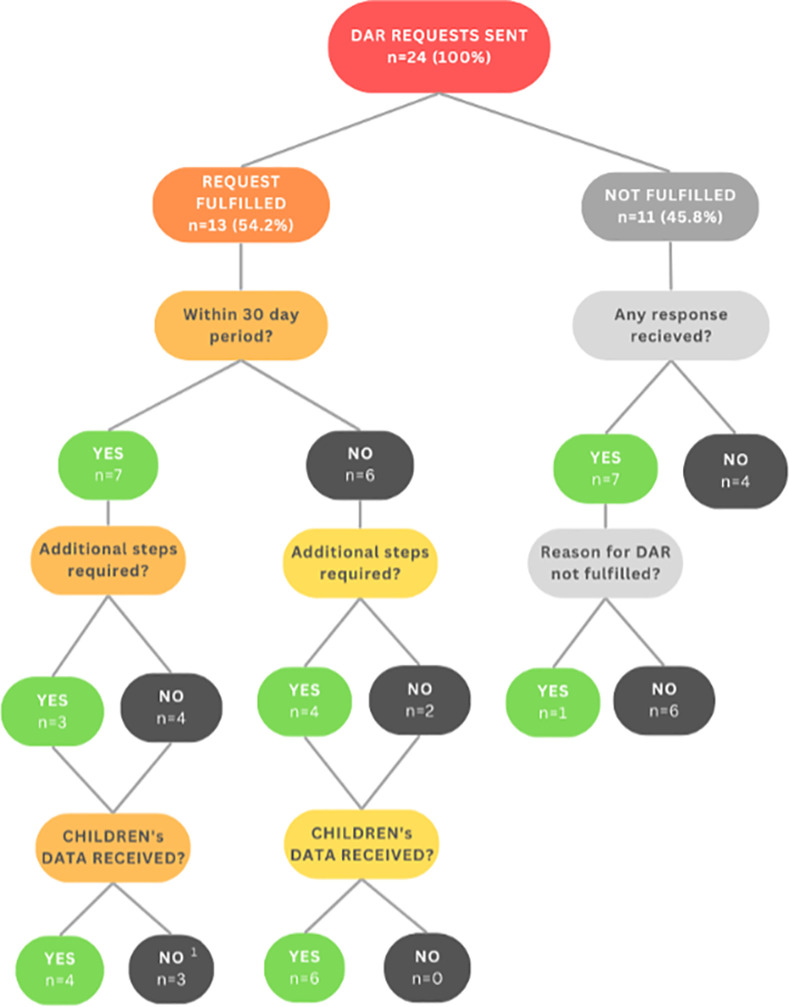
A Summary Of The Dar Process And Response Rates From Food Companies.^1^ Alternative outcomes to receiving children’s data included receiving the parent’s account data instead (n = 1), or the company closing the child’s account without providing data (n = 2).

The study sample included 11 participants (mean age: 10.6 years; range: 9–12 years) who, in combination, submitted (or had a parent/guardian submit on their behalf) 24 DARs for the 5 fast-food company mobile apps examined. Between 3 (Company 2) and 6 (Companies 1 and 5) DARs were submitted per app. **Fig 2** and **Table 2** summarize the DAR process and associated results. Overall, companies fulfilled 54.2% (n = 13) of the submitted DARs. Two companies (Companies 1 and 5) fulfilled 83.3% of the DARs submitted to them. Companies 2 and 4 did not fulfill any requests. Less than one-third (n = 7, 29.2%) of DARs were fulfilled with the 30-day period mandated by PIPEDA, of which 3 (42.3%; 12.5% of total DARs) required further procedures for fulfillment, and 4 (57.1%; 16.7% of total DARs) were successful in obtaining participants’ data. Company 1 did not respond to any DARs within the 30-day period required by PIPEDA, while Companies 2 and 4 did not respond to any requests throughout the study period.

**Table 2:** A summary of the DAR process and results, presented overall and by food company.

		COMPANY 1 (n=6)^1^	COMPANY 2 (n=3)	COMPANY 3 (n=5)	COMPANY 4 (n=4)	COMPANY 5 (n=6)	TOTAL (n=24)
**FULFILLED DARS**		5 (83.3)^2^	0 (0.0)	3 (60.0)	0 (0.0)	5 (83.3)	13 (54.2)
**DARS FULFILLED WITHIN THE REQUIRED 30-DAY PERIOD** ^3^		0 (0.0)	N/A	2 (40.0)	N/A	5 (83.3)	7 (29.2)
Additional steps required to fulfill DAR	Yes	N/A	N/A	2 (40.0)	N/A	1 (16.7)	3 (12.5)
	No	N/A	N/A	0 (0.0)	N/A	4 (66.7)	4 (16.7)
DAR results in the receipt of children’s data	Yes	N/A	N/A	1 (20.0)	N/A	3 (50.0)	4 (16.7)
	No	N/A	N/A	1 (20.0)	N/A	2 (33.3)	3 (12.5)
**DARS FULFILLED OUTSIDE OF THE REQUIRED 30-DAY PERIOD** ^3^		5 (83.3)	N/A	1 (20.0)	N/A	0 (0.0)	6 (25.0)
Additional steps required to fulfill DAR	Yes	3 (50.0)	N/A	1 (20.0)	N/A	N/A	4 (16.7)
	No	2 (33.3)	N/A	0 (0.0)	N/A	N/A	2 (8.3)
DAR results in the receipt of children’s data	Yes	5 (83.3)	N/A	1 (20.0)	N/A	N/A	6 (25.0)
	No	0 (0.0)	N/A	0 (0.0)	N/A	N/A	0 (0.0)
**UNFULFILLED DARS**		1 (16.7)	3 (100.0)	2 (40.0)	4 (100.0)	1 (16.7)	11 (45.8)
Response received from the company	Yes	0 (0.0)	3 (100.0)	2 (40.0)	2 (50.0)	0 (0.0)	7 (29.2)
	No	1 (16.7)	0 (0.0)	0 (0.0)	2 (50.0)	1 (16.6)	4 (16.7)
Reason for the DAR not being fulfilled (*For the n=7 requests that received any response)*	Yes	N/A	1 (33.3)	0 (0.0)	0 (0.0)	N/A	1 (4.2)
	No	N/A	2 (66.7)	2 (40.0)	2 (50.0)	N/A	6 (25.0)

^1^ *Number of DARs sent;*

^2^ *n (%);*

^3^ *Required as per PIPEDA [* 31 *].*

In total 25.0% (n = 6) DARs were fulfilled outside of the 30-day period required by PIPEDA; 5 of these requests were sent to Company 1, while the remaining request was to Company 3. Four of the requests fulfilled beyond the 30-day period (66.7%; 16.7% of total DARs) required further procedures for fulfillment (e.g., completing forms or sending additional emails) and all requests were successful in obtaining participants’ data. Nearly half (n=11, 45.8%) of DARs remained unfulfilled by the study’s end date (i.e., 30 days after the follow-up email or 60 days after submission of the initial DAR). Of these unfulfilled requests, 7 had been responded to by the company (63.6%; 29.2% of total requests), and 1 request was provided with justification for the unfulfillment.

**Table 3** summarizes the additional procedures that food companies required for DAR fulfilment, and outlines the justifications provided for unfulfilled requests. All companies responded to ATI submissions by requesting additional information from participants, with 5 companies seeking evidence of the parent-child relationship to ensure children’s data were only being shared with authorized individuals. Some companies (2, 3, 4 and 5) also requested age verification of the child (e.g., by providing a birth certificate). DAR responses also varied within companies. For instance, three distinct responses were provided to DARs received by Company 5. Despite almost 50% of DARs being unfilled, only one unfulfilled request was accompanied with justification for doing so (by Company 2), with the company stating that more time would be required to properly format the information.

**Table 3:** A summary of the observed additional procedures required to fulfill DARS and justifications for unfulfillment.

	Additional steps required to fulfill DAR	Reasons for ATI not being fulfilled
COMPANY 1	The company requested proof of the parent-child relationship by asking the child to email the privacy officer within 30 days to confirm that their parent had permission to access the child’s personal data, while copying (“cc’ing”) that parent on the email.	Company did not provide an explanation.
COMPANY 2	The company requests that parents provide proof of their child’s age and their relationship with the child (e.g., birth certificate) to confirm they were authorized to receive the child’s personal data. A short-form birth certificate submitted by one of the parent participants was said to not fulfill this requirement and further evidence of the parent-child relationship was requested by the company. In the end, no DARs were fulfilled.	The company stated that more time would be required than initially expected to format the information properly.
COMPANY 3	The company requested that some parents fill out an online form to confirm their identity. Parents were also asked to provide documentation to verify their child’s identity, their relationship to the child, and the child’s age (e.g., in the form of a birth certificate).	Company did not provide an explanation.
COMPANY 4	The company requested that parents confirm their child’s birth date and informed them that further documentation would be required to verify their child’s identity and their relationship to the child. In the end, no DARs were fulfilled.	Company did not provide an explanation.
COMPANY 5	The company responded to some parents’ requests by redirecting their request to the Customer Care team, stating that the Data Privacy Team (where the initial request was sent) does not have the authority to manage or delete personal data related to user accounts. After contacting Customer Care, one parent participant was provided with a phone number and received information about their case via phone, having been informed that further information would not be provided via email. Other parent participants were informed that the company’s services were not meant for children under 13 years of age, as indicated in their Privacy Notice and Terms of Use. The company asked parents to verify their child’s age so they could terminate the account. In such instances, no DARs were fulfilled.	Company did not provide an explanation.

A summary of the personal data collected on children (based on data provided by companies in response to DARs) is provided in **Table 4**. Sociodemographic data on child participants (e.g., name, email address, country of residence) were collected by Companies 1, 3 and 5. Information about mobile orders placed (e.g., data and time or purchase, and total order cost) and promotional offers that were available to the participant (e.g., offer name, dates during which offer was valid, description of offer, etc.) were also collected by Companies 1 and 5. Additionally, communications to the participant (e.g., push notifications) were monitored by Company 1. Details about participants’ subscriptions to rewards programs, use of gift cards or in-store Wi-Fi, and app use analytics (e.g., date and time of app usage, operating system of digital device used, total clicks in the app, etc.) were tracked by Company 5.

**Table 4:** A summary of the personally-identifiable information and other details collected by the 5 food companies analyzed, based on receipt of children’s data^1^.

	COMPANY 1	COMPANY 3	COMPANY 5	
**Personally-identifiable or other information collected** ^**2**^	**Profile information:** • Date and time of account creation • Country code • First name • Last name • Language • Postal code • Activity status • Email **Communications with participant:** • Channel • Whether it was sent or opened • Date and time sent • Device operating system **Orders:** • Date and time order placed • How many offers were applied • Total net cost • Total gross cost • Tax amount • Payment method • Channel (front counter, drive-through) **Offer details:** • Date and time • Description of offer	• First name • Last name • Country • Entity ID • Email • Phone number • Language	**Profile information:** • Email • First name • Last name • Birth day • Birth month • Address • City • Province • Zip code • Country • Phone number **Rewards, subscription, Wi-Fi registration information** • Rewards member status • Rewards creation date • Registered Wi-Fi • Email opt-in • Email opt-in source **Gift Cards** • Gift card number **Business Gift Cards** • Transaction date • Quantity • Amount **Purchase Transactions** • Date and time order placed • Store name • Order total charged • Item name	**Rewards Transactions** • Date earned • Points earned • Point types (points) **Promotions** • Promotion name • Start date • Expiration date • Redemption date • Status **Favourites** • Product name **Wi-Fi Device Registration** ^**3**^ • First name • Last name • Customer email address **Google Analytics** ^**4**^ • Visit date and time • Visit number • Country visited from • Operating system • Operating system version • App version • Total clicks • Internet service provider

^1^ *Companies 2 and 4 were not included in table, as no DARs were fulfilled;*

^2^ *Information categories and names reported as provided by the food company;*

^3^ *Information entered when signing up to use in-store Wi-Fi;*

^4^ *Information collected within the app to enhance functionality for app users.*

## Discussion

This study found that current Canadian privacy policies are not sufficiently protecting children’s digital data, nor are these policies preventing children from using food company mobile apps, despite the apps being directed at older age groups.

Although the privacy policies of some food companies specified that their mobile apps were not intended for users under 13 years of age, more than half (54%) of the companies in this sample did not specify any age restrictions for their apps. Notably, none of the apps included mandatory age verification for users. Previous research indicates food company mobile apps are popular among Canadian children, and findings of the present study suggest that food companies’ current privacy policies are not limiting children’s usage of their apps [25]. Moreover, 92.3% of the sampled food companies stated in their privacy policies that their mobile apps collect personally identifiable information from users (e.g., name, address, payment details). Of these companies, 89% did not specify whether their data collection process differentiates between child and adult users. In combination, these findings highlight the vulnerability of children to the collection and exploitation of their personal data for digital food marketing purposes. Most company privacy policies (81%) did describe a process for handling data inadvertently collected on children through their apps, whereby the company would erase such data or consider requests from parents to have the data erased. Additionally, the privacy policies of most food companies described procedures for handling users’ personal information that aligned with requirements established in PIPEDA (e.g., listing contact details for a privacy officer, and outlining users’ rights to request data access and amend or delete their personal data). Nonetheless, it is concerning that the policies of 5 companies did not reference a process for handling or deleting children’s data.

As part of this study, we also examined what data food companies are collecting on child users of their mobile apps. The extent of children’s digital data collected from fast-food apps was found to be unclear, as responses to DARs were inconsistent across companies. It was, however, found that personal data is being collected on child users of food company mobile apps (e.g., name, birth date, country of residence, preferred language, email address). Some of these apps also collect data on children’s food preferences and purchasing habits (e.g., preferred foods and food order history, purchases in response to previous in-app promotions). In more extreme cases, additional analytic data was collected, such as the mobile operating system of the app user’s device, provider of the internet used to access the app, frequency of app visits and in-app clicks, and/or the number and type of app notifications received and viewed by the user.

Although the data collected on child mobile app users in this study seemingly aligns with the companies’ privacy policies, the type of data collected varied between companies. In addition, it is unclear how children’s data may be used to inform targeted marketing practices; such an investigation was beyond the scope of this study but should be examined in future research. Furthermore, the types and amounts of data being collected on child app users in this study are likely underestimations, as there was no response to nearly half of the DARs sent (including all DARs to Companies 2 and 4). The privacy policies of several companies indicated that children’s personal data collected through the app could be anonymized, aggregated and transmitted to third parties; however, the responses to DARs did not indicate how the data were analyzed or shared. Previous research has demonstrated that child-directed mobile apps often share data with third parties [26]; subsequent studies should examine children’s data handling and transmission on food company mobiles apps to help fill this knowledge gap.

Despite these concerns, it is important to consider that all food companies to which DARs were submitted indicated their app was not meant for children under 13 years of age. Thus, by consenting to the privacy policy and terms of service, children are stating that they are at least 13 years-old and indicating to companies that they have permission to collect their personal data. Companies may assert that this indication of permission provides them with legal protections from liability for collecting data on children. Regardless of the validity of such an assertion the ability for children to enter into this kind of an agreement raises several concerns for children’s privacy and wellbeing. First, children are using mobile apps that were not meant for them, as evidenced by the large number of children aged 9-12 years in this study with accounts on food company apps already downloaded to their devices. Children also spend a considerable amount of time on social media accounts (e.g., TikTok, Instagram, YouTube), despite company policies requiring users to be at least 13 years-old [35]. Moreover, food company privacy policies often make no reference to adolescents aged 13-18 years, leaving them susceptible to marketing messages. Their still-developing cognitive and decision-making skills – combined with spending large amounts of time online – leave adolescents vulnerable to digital marketing of unhealthy foods and beverages [36]. Collecting personal data on adolescent mobile app users, though not the subject of investigation in this study, may potentially provide food companies with increased opportunities for targeted marketing of less healthy foods to this age group as well and should be studied.

Although the cohort study aimed to explore the types of data that food company mobile apps are collecting on children, it also sheds light on how children’s data is obtained. The policy scan found that most DAR processes stated in the privacy policies of the companies in this sampled aligned with PIPEDA requirements, yet our results also indicate these policies do not consistently translate to actions. Responses to DARs were inconsistent within and across companies, and two companies did not fulfill any requests. Furthermore, additional steps or details not described in the companies’ policies were often required to obtain participants’ data. For example, parents in this sample often had to (ironically) submit additional personal information and supporting documentation (e.g., birth certificates, passports) to verify their identity and that of their child, and their child’s parentage, while other parent participants were asked to complete more forms, consult other websites, or send further emails. Undergoing these additional steps did not necessarily result in access to the child’s data. The number and extent of additional steps required also varied by food company, with as many as three separate procedures needed to secure access to children’s data from one food company. Furthermore, although PIPEDA indicates that food companies must inform individuals if their DAR cannot be fulfilled in 30 days [31], none of the sampled companies met this deadline. Only one of the 11 unsuccessful DARs submitted in this study was accompanied with an explanation for the lack of fulfilment (e.g., requiring more time to complete the request), and the participant had still not received their requested data from that company by the time of study completion. Ten other DARs (nearly half of all submitted requests) were never fulfilled or communicated about by the companies, suggesting that many food companies do not fully abide by PIPEDA in handling data requests from users.

### Future considerations

In light of digital media continuously evolving and companies using a growing number and variety of digital marketing strategies to target and exploit children, there is a need for governments to re-evaluate the effectiveness of their policies and regulations concerning children’s digital privacy. Findings of this study suggest strengthening of these policies and regulations is warranted and that restrictions on food marketing should extend to all digital media via which children are exposed, including food company mobile apps. Digital privacy regulations should differentiate between adults and children, and should provide heightened protections for all children under 18 years of age (as per the United Nations and World Health Organization age definitions) [30,37–39]. Furthermore, implementing a federally mandated procedure for age verification of digital media users could reduce the likelihood that companies would inadvertently collect young children’s data, though any such process would need to be demonstrably compliant with human rights or *Charter* protections. While strengthening of food companies’ individual digital privacy policies is warranted, it does not negate the need for federal regulations to ensure consistent good practices across companies, including in how they respond to requests for children’s information from parents or guardians. Moreover, policies should extend beyond digital marketing that is “child-directed” or “child-targeted” since these definitions often exclude less traditional digital media platforms via which children are frequently exposed to unhealthy food marketing, including the food company mobile apps examined in this study. Food marketing restrictions should encompass all digital environments where children are present.

### Strengths & limitations

This work provides a novel evaluation of Canadian privacy policies for the mobile apps of leading food service companies and the data being collected on child users of these apps. Study strengths include the analysis of all publicly available privacy policies and terms of service documents published by food companies, and our use of existing research methods to guide our approach to data collection. Our research also provides insight into the procedures required for accessing users’ personal data, which had not previously been examined for food companies in Canada. Nonetheless, this study has some important limitations. First, the research team relied on participants for timely communication with food companies and to share all study-related email exchanges with the researchers. Accordingly, delays in participant responses to food companies may have contributed to the lack of ATI fulfillment within the 30-day period; researchers were not able to access all email exchanges. Nonetheless, communications between participants and food companies were consistent and guided by email templates that were pre-drafted by researchers who conducted similar studies in other industries [34,40]. The accuracy and completeness of data collection was also bolstered by independent review by a second researcher. Other limitations were that this research did not assess the privacy policies of Android applications and whether they have similar, or divergent privacy implications for child users than those of the apps accessed via the iOS App Store. Moreover, it was out of scope for this study to conduct a technical assessment of the included applications, for the purpose of determining whether the responsive information to DARs was fulsome or not. This project therefore presumed that the materials provided by the companies was comprehensive.

## Conclusions

The study showed that the current privacy policies of major fast-food and dine-in restaurant companies in Canada do not sufficiently prevent companies from collecting children’s data, nor do these policies appropriately limit children’s use of the companies’ mobile applications. This work found that these apps are collecting children’s personal data, although the full extent to which the data is being collected is unclear, as most companies did not respond to DARs. In light of the evidence characterizing children’s exposure to unhealthy food marketing on digital media and the use of personal data to deliver targeted digital marketing, further research is warranted to improve our understanding of food companies’ practices concerning the collection and use of digital data in marketing [21,22,36]. Overall, this research highlights gaps in food company policies concerning children’s personal data and digital privacy in Canada. Food marketing restrictions should incorporate a child rights-based approach to help limit children exposure to targeted digital marketing of unhealthy foods.

## Supporting information

S1 Fig 1 Questions used to guide the analysis of the Data Access Request (DAR) process. (DOCX)

S1 Table Questions used to guide the analysis of food company privacy policies and terms of service agreements. (DOCX)

S2 Table A detailed summary of mobile app privacy policies and/or terms of service agreements from the top food companies in Canada (n=26) (DOCX)
