# Supplementary figures and images for: Children’s digital privacy on fast-food and dine-in restaurant mobile applications
Source: PLOS Digit Health. 2025 Feb 5;4(2):e0000723. doi: 10.1371/journal.pdig.0000723 (PMC11798428; doi:10.1371/journal.pdig.0000723)

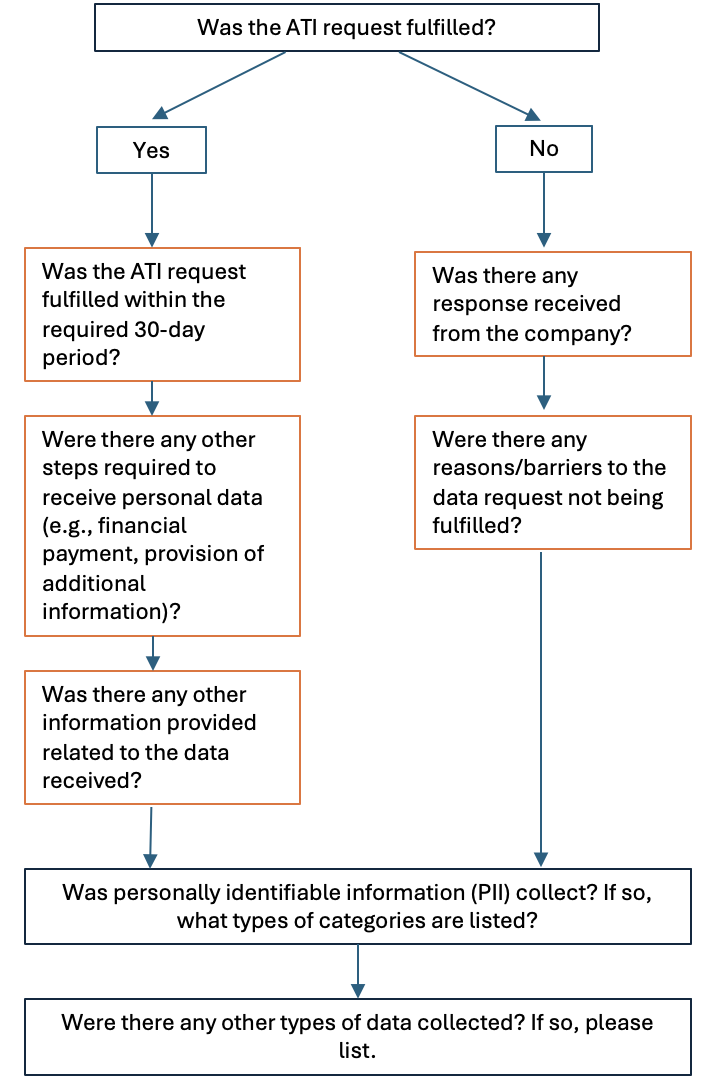


**S1 Fig 1.** Questions used to guide the analysis of the Data Access Request (DAR) process.

Supplement: S1 Fig 1 — (DOCX) [file pdig.0000723.s001.docx]
