# Supplementary material for: Children’s digital privacy on fast-food and dine-in restaurant mobile applications
Source: PLOS Digit Health. 2025 Feb 5;4(2):e0000723. doi: 10.1371/journal.pdig.0000723 (PMC11798428; doi:10.1371/journal.pdig.0000723)
Supplement: S1 Table — (DOCX) [file pdig.0000723.s002.docx]

**S1 Table.** Questions used to guide the analysis of food company privacy policies and terms of service agreements.

| **Questions concerning company privacy policies/terms of service/end user license agreements** | Is there a link to a privacy policy on the app log in, ‘make an account’ page or homepage? If not, where was the privacy policy located? (Please provide link in Excel sheet). |
| --- | --- |
|  | Is the privacy policy specific to the mobile application or more broad (e.g., also pertaining to the company website)? |
|  | Is there a reference to compliance with: national privacy laws, international guidelines, or self-regulatory instruments from associations? |
|  | Is there a statement concerning which nation/court proceedings must go through? |
|  | Is there a reference in the privacy policy to the Terms of Service or End User License Agreement, and vice versa? |
|  | Does the privacy policy and/or Terms of Service or End User License Agreement indicate the age of the intended user of the mobile application? |
|  | Is there information about when the privacy policy was last updated? Is it dated? Can one access previous versions? |
|  | Does the company reserve the right to change the privacy policy or other public policy documents that might establish terms around the collection, use, or processing of personal information without notification? If notification is promised, under what conditions are users notified? Is notification promised to all persons whose personal information has been collected? What are the terms of accepting the new policy? |
| **Questions concerning accessing information about a company’s policies** | Is there a contact to a privacy officer listed? (For top 5 apps, please list) |
|  | Is there a description/discussion of who a person can complain to if they’re unsatisfied with the information/processes laid out in an organization’s public facing documents? |
|  | Is there a process for deleting one’s information (i.e., a “Right to forget)? |
|  | Is there any statement regarding what happens if data was inadvertently collected on children?  Do you have to be a customer or active user of a company’s products to make use of any stated procedures (e.g., right to access or delete information) |
| **Questions about a company’s collection of personal (or personally identifying) information** | Are there details of the specific kinds of Personally Identifiable Information (PII) which are collected? If so, what types of categories are listed? |
|  | Is there any distinction made between sensitive and non-sensitive PII? |
|  | Is any distinction made between information pertaining to children or adults? |
|  | Is there any age verification process (e.g., entering date of birth)? |
|  | Does the company require that certain information is provided, as a precursor to signing up for the service or acquiring products from the company? If so, what is asked for or collected? (e.g., during the account registration process) |
| **Questions concerning data security** | Are commitments made to the security of PII? |
|  | Are commitments made to the encryption or deidentification of data? |
|  | Is there a note that users or government bodies are alerted if a data breach occurs? Are all persons who have their information disclosed notified, or only those contracting with the company? |
| **Questions concerning access and correction rights** | Is there a distinction between “users” and “targeted persons” when it comes to access and correction rights? |
|  | Are commitments made to allow the access of either PII or non-PII? |
|  | Are commitments made to all correction of either PII or non-PII? |
|  | Are procedures for access and correction specified? For persons contracting with the company? For persons targeted by the company’s products or services? |
|  | Is there a stated monetary cost associated with gaining access to one’s PII or non-PII? |
