# Supplementary material for: Children’s digital privacy on fast-food and dine-in restaurant mobile applications
Source: PLOS Digit Health. 2025 Feb 5;4(2):e0000723. doi: 10.1371/journal.pdig.0000723 (PMC11798428; doi:10.1371/journal.pdig.0000723)
Supplement: S2 Table — (DOCX) [file pdig.0000723.s003.docx]

**S2 Table.** **A detailed summary of mobile app** **privacy policies and/or terms of service agreements from the top food companies in Canada (n=26)^1^**

| **Questions concerning company privacy policies and/or terms use agreements** | | **n (%)** | **Notes** |
| --- | --- | --- | --- |
| 1. Is there a link to a privacy policy on the app login, ‘make an account’ page or homepage? | Yes | 26 (100) | 26 food companies have a link to the privacy policy on their website homepage.  21 food companies have a link to the privacy policy on their app login, ‘make an account’, or homepage. |
|  | No | 0 (0) |  |
| 1. Is the privacy policy specific to the mobile application or apply more broadly^2^ | Mobile application | 21 (80.7) |  |
|  | Website | 25 (96.1) |  |
| 1. Is there a reference to compliance with: national privacy laws, international guidelines, or self-regulatory instruments from associations? | Yes | 12 (46.2) | Personal information may be stored outside of the province of residence and/or outside of Canada and be subject to the laws of those jurisdictions. |
|  | No | 14 (53.8) | 13 food companies only refer to “applicable law”, but do not specify which laws. |
| 1. Is there a statement concerning which nation/court proceedings must go through? | Yes | 4 (15.4) | For example, parties consent to the jurisdiction of the courts of Montreal, Québec. Or disputes will be resolved by arbitration in Toronto, Ontario. |
|  | No | 22 (84.6) |  |
| 1. Is there a reference in the privacy policy to the Terms of Service or End User License Agreement, and vice versa? | Yes | 25 (96.2) | 6 food companies only refer to the privacy policy from the terms of service.  1 food company only refers to the terms of service from the privacy policy. |
|  | No | 1 (3.8) |  |
| 1. Does the privacy policy indicate the age of the intended user of the mobile application? | Yes | 12 (46.2) | 10 food companies specify that their app is not intended for children under age 13. |
|  | No | 14 (53.8) |  |
| 1. Is there information about when the privacy policy was last updated? | Yes | 24 (92.3) |  |
|  | No | 2 (7.6) |  |
| 1. Does the company reserve the right to change the privacy policy or other public policy documents that might establish terms around the collection, use, or processing of personal information without notification? | Yes | 3 (11.5) |  |
|  | No | 23 (88.5) | Food companies typically indicate changes to their policy by updating the ‘last modified date’. These changes to the Privacy Policy become effective when the updated policy is posted. |
| **Questions about accessing information about a company’s policies** | | **n (%)** | **Notes** |
| 1. Is there a contact to a privacy officer listed? | Yes | 26 (100) | Food companies typically provide a mailing address, email, and/or phone number for questions about their privacy policy. |
|  | No | 0 (0) |  |
| 1. Is there a description/discussion of who a person can complain to if they’re unsatisfied with the information/processes laid out in an organization’s public facing documents? | Yes | 25 (96.2) | Food companies typically welcome questions, comments, and requests regarding their privacy policy. |
|  | No | 1 (3.8) |  |
| 1. Is there a process for deleting one’s information? | Yes | 15 (57.7) | Food companies may retain certain information if required by law. |
|  | No | 11 (42.3) |  |
| 1. Is there any statement regarding what happens if data was inadvertently collected on children? | Yes | 21 (80.8) | 11 food companies commit to deleting information collected from children under age 13 in the event they become aware of it.  9 food companies allow parents whose child has provided personal information to ask for it to be deleted. |
|  | No | 5 (19.2) |  |
| 1. Do you have to be a customer or active user of a company’s products to make use of any stated procedures? | Yes | 0 (0) |  |
|  | No | 26 (100) |  |
| **Questions about company’s collection of personal information** | | **n (%)** | **Notes** |
| 1. Are there details of the specific kinds of PII which are collected? | Yes | 24 (92.3) | For example, PII may include name, email address, location, birthday, gender, street address, mobile phone number, payment information, etc. |
|  | No | 2 (7.7) |  |
| 1. Is there any distinction made between sensitive and non-sensitive PII? | Yes | 0 (0) |  |
|  | No | 26 (100) |  |
| 1. Is any distinction made between information pertaining to children or adults? | Yes | 3 (11.5) | 1 food company states that it will not collect more detailed information from a child age 12 and younger without the consent of a parent.  1 food company identifies portions of its service that are appropriate for a child under age 13 where personal information will not be collected without compliance with COPPA (i.e., American children’s privacy law). |
|  | No | 23 (88.5) |  |
| 1. Is there any age verification process (e.g., entering date of birth)? | Yes | 0 (0) |  |
|  | No | 26 (100) | Some food companies allow for optional disclosure of age, while others state that by creating a profile you confirm that you are at least a certain age. |
| 1. Does the company require that certain information is provided, as a precursor to signing up for the service or acquiring products from the company? | Yes | 26 (100) | This information includes a combination of name, email, postal code, phone number, etc. |
|  | No | 0 (0) |  |
| **Questions around the security of collected information** | | **n (%)** | **Notes** |
| 1. Are commitments made to the security of PII? | Yes | 21 (80.8) | Food companies describe physical, electronic, and managerial procedures to protect PII against loss, theft, and unauthorized access, use, modification, and disclosure. |
|  | No | 5 (19.2) |  |
| 1. Are commitments made to the encryption or deidentification of data? | Yes | 10 (38.5) | PII may be anonymized and/or aggregated and stored to analyze trends. |
|  | No | 16 (61.5) |  |
| 1. Is there a note that users or government bodies are alerted if a data breach occurs? | Yes | 3 (11.5) | In the event of a breach of data security, 3 food companies will take reasonable steps to investigate the situation and, where appropriate, notify individuals whose information may have been compromised and take additional steps, in accordance with any applicable laws and regulations. |
|  | No | 23 (88.5) |  |
| **Questions about company’s collection of personal information** | | **n (%)** | **Notes** |
| 1. Is there a distinction between “users” and “targeted persons” when it comes to access and correction rights? | Yes | 0 (0) |  |
|  | No | 26 (100) |  |
| 1. Are commitments made to allow access to either PII or non-PII? | Yes | 21 (80.8) | 21 food companies specify the right to access personal information. |
|  | No | 5 (19.2) | 1 food company specifies this only for California and EU residents. |
| 1. Are commitments made to allow correction of either PII or non-PII? | Yes | 21 (80.8) | 21 food companies allow users to request their personal information to ensure its accuracy. |
|  | No | 5 (19.2) | 1 food company specifies this only for California and EU residents.  1 food company specifies that if personal information is directly supplied by the user it is assumed to be correct. |
| 1. Are procedures for access and correction specified? | Yes | 22 (84.6) | Users are to contact the privacy officer. |
|  | No | 4 (15.4) |  |
| 1. Is there a stated monetary cost associated with gaining access to one’s PII or non-PII? | Yes | 0 (0) |  |
|  | No | 26 (100) |  |

^1^Data collected in November 2022; ^2^Percentages add up to greater than 100% as companies could have presented more than one option.
